# Supplementary material for: Informing the Design of Data Visualization Tools to Monitor the COVID-19 Pandemic in Portugal: A Web-Delphi Participatory Approach
Source: Int J Environ Res Public Health. 2022 Sep 2;19(17):11012. doi: 10.3390/ijerph191711012 (PMC9517757; doi:10.3390/ijerph191711012)
Supplement: Supplementary file 1 [file ijerph-19-11012-s001.zip › ijerph-1817361-supplementary.pdf]

Supplementary Material S1

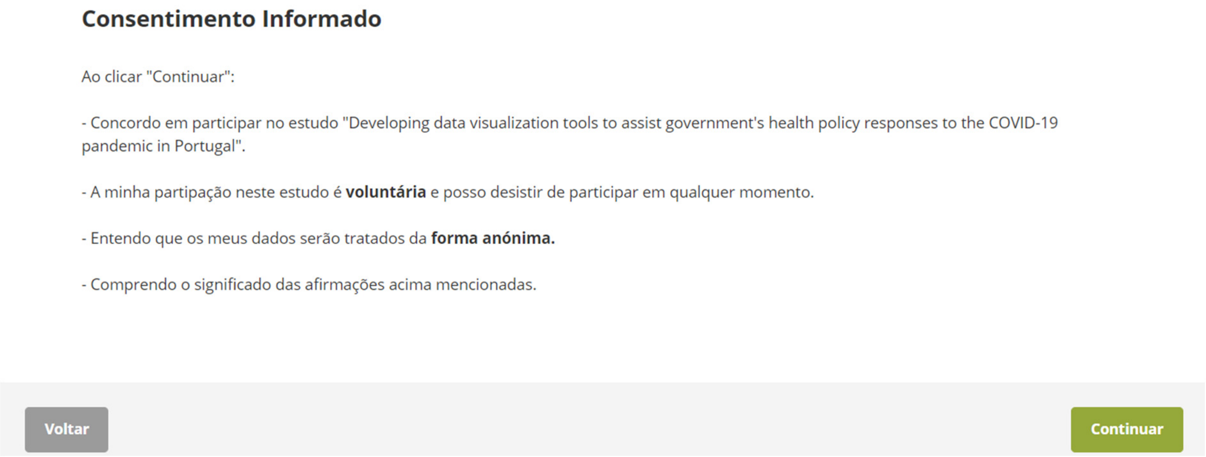

Figure S1. Consent agreement screen. Trans. Consent agreement. By clicking "Continue": - I agree to participate in the study "Developing data visualization tools to assist government's health policy responses to the COVID-19 pandemic in Portugal". - My participation in this study is voluntary and I can opt out at any time. - I understand that my data will be treated anonymously. - I understand the meaning of the above statements.

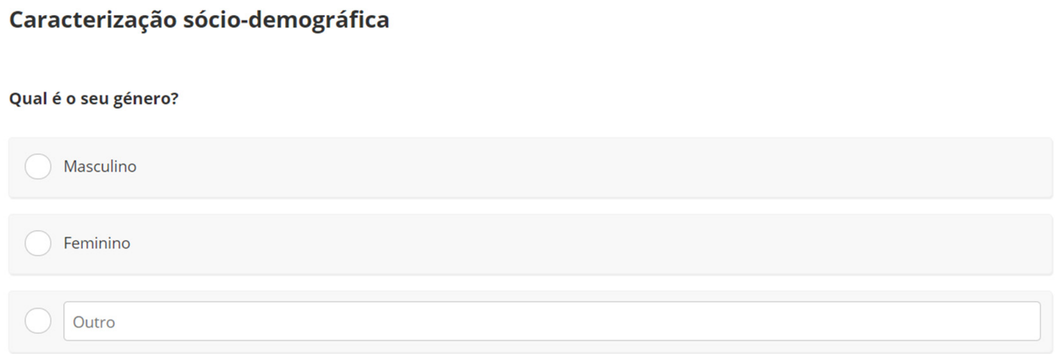

Figure S2. Socio-demographic characterization screen. Trans. What is your gender? – Male; - Female - Other.

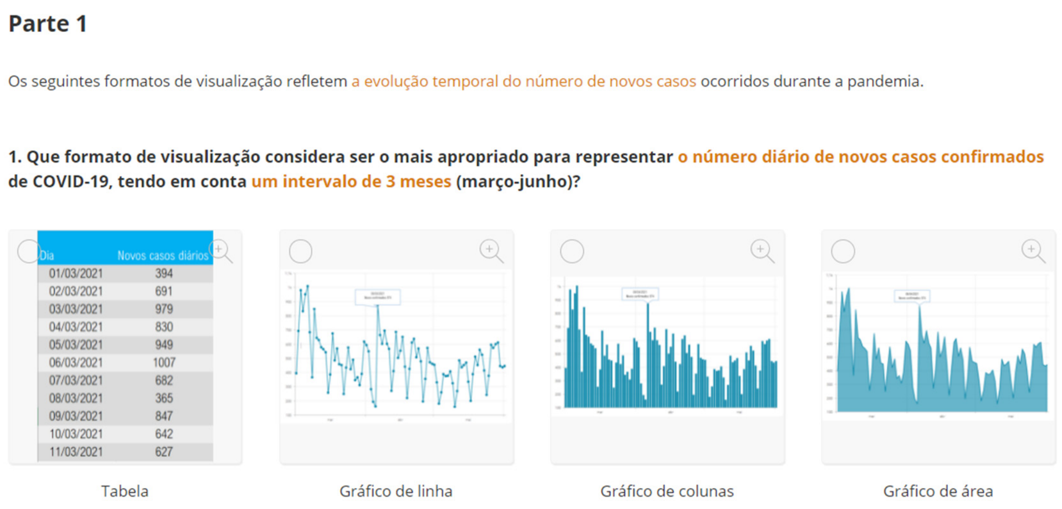

Figure S3. Example of screen in Part 1. Trans. The following visualization formats reflect the temporal evolution of the number of new cases that occurred during the pandemic. What visualization format

do you consider to be the most appropriate to represent a daily number of new confirmed COVID-19 cases, considering three-month time interval (March-June)? – Table; - Line Chart; - Column Chart; - Area Chart.

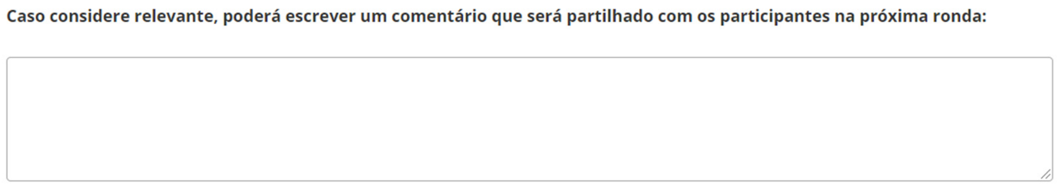

Figure S4. Example of comment's input area. Trans. If you consider it relevant, you can write a comment that will be shared with the participants in the next round.

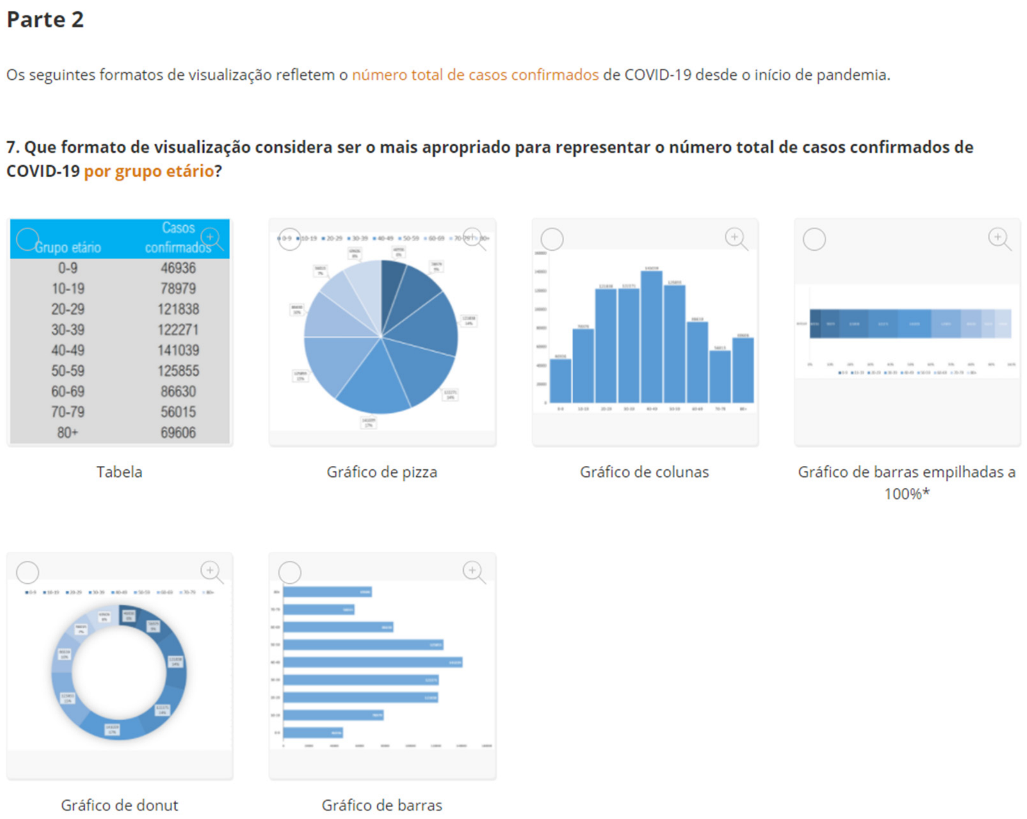

Figure S5. Example of screen in Part 2. Trans. What visualization format do you consider to be the most appropriate to represent a total number of confirmed COVID-19 cases by age group? – Table; - Pie Chart; - Column Chart; - 100% Stacked Bar Chart; Donut Chart; Bar Chart.

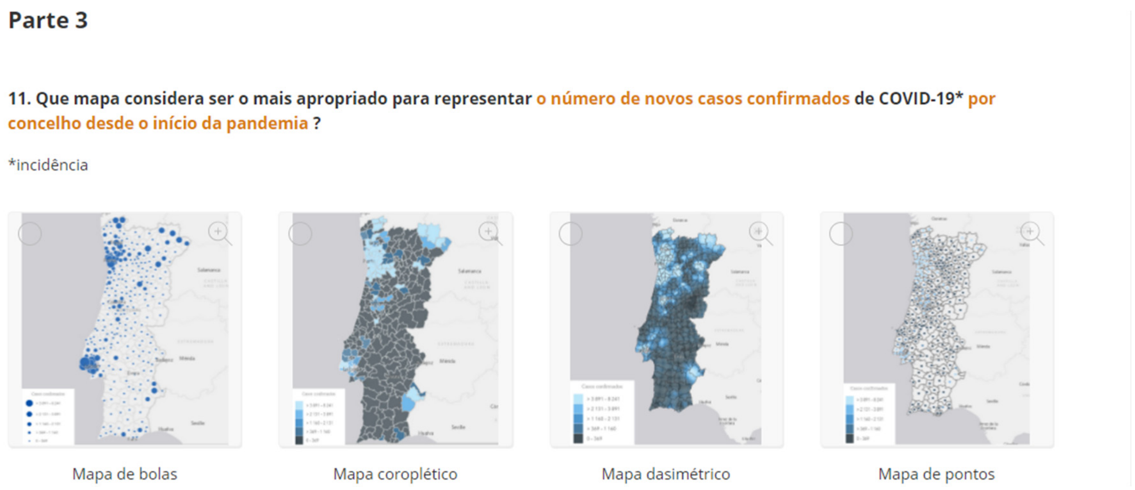

Figure S6. Example of screen in Part 3. Trans. What map do you consider to be the most appropriate to represent a number of new confirmed COVID-19 cases per municipality (incidence) since the beginning of pandemic? – Bubble Map; - Choropleth Map; - Dasymetric Map; Point Map.

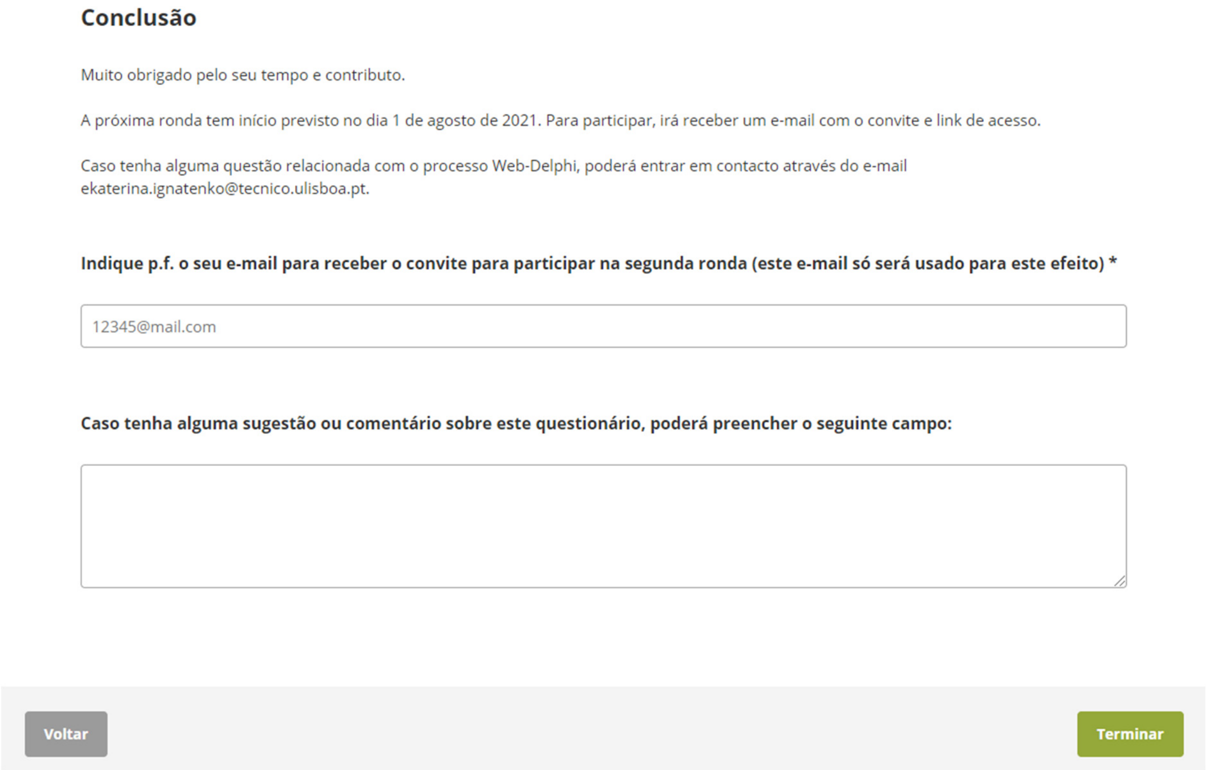

Figure S7. Conclusion screen. Trans. Thank you very much for your time and contribution. The next round is scheduled to start on August 1, 2021. To participate, you will receive an email with an invitation and access link. If you have any questions related to the Web-Delphi process, you can contact us via e-mail ekaterina.ignatenko@tecnico.ulisboa.pt. Please enter your e-mail address to receive the invitation to participate in the second round (this e-mail will only be used for this purpose). If you have any suggestions or comments about this questionnaire, you can fill in the following field.

Supplementary Material S2

Table S1. Questionnaire responses for the first round.

№	Gender	Age group	Highest education level	Have the professional activity related with health sector	Are familiar with COVID-19 dashboards	Question 1	Question 2	Question 3	Question 4
1	Male	20 - 29 years	Bachelor’s degree	Don’t exercise any professional activity	Yes	Column Chart	Line Chart	Area Chart	Area Chart
2	Male	20 - 29 years	Bachelor’s degree	Yes	No	Line Chart	Column Chart	Line Chart	Column Chart
3	Female	20 - 29 years	Master’s degree	Yes	Yes	Area Chart	Table	Area Chart	Table
4	Female	20 - 29 years	Master’s degree	No	No	Line Chart	Table	Column Chart	Column Chart
5	Female	20 - 29 years	Master’s degree	Yes	No	Area Chart	Area Chart	Line Chart	Line Chart
6	Female	20 - 29 years	Master’s degree	No	No	Line Chart	Column Chart	Line Chart	Area Chart
7	Male	20 - 29 years	Bachelor’s degree	No	Yes	Column Chart	Line Chart	Area Chart	Column Chart
8	Female	20 - 29 years	Bachelor’s degree	No	No	Area Chart	Column Chart	Line Chart	Column Chart
9	Male	20 - 29 years	High School	No	No	Area Chart	Line Chart	Column Chart	Line Chart
10	Female	20 - 29 years	Techno-professional School	No	Yes	Column Chart	Table	Column Chart	Column Chart
11	Male	20 - 29 years	Master’s degree	Yes	Yes	Line Chart	Table	Column Chart	Column Chart
12	Male	20 - 29 years	Bachelor’s degree	No	Yes	Line Chart	Line Chart	Line Chart	Line Chart
13	Male	30 - 39 years	Master’s degree	No	Yes	Table	Table	Table	Table
14	Female	0 - 19 years	High School	Don’t exercise any professional activity	No	Line Chart	Column Chart	Area Chart	Column Chart
15	Female	20 - 29 years	Bachelor’s degree	Yes	Yes	Line Chart	Column Chart	Line Chart	Column Chart
16	Female	20 - 29 years	Master’s degree	No	Yes	Area Chart	Column Chart	Area Chart	Area Chart
17	Male	20 - 29 years	Master’s degree	No	Yes	Area Chart	Column Chart	Line Chart	Line Chart
18	Male	20 - 29 years	Bachelor’s degree	Don’t exercise any professional activity	Yes	Line Chart	Line Chart	Column Chart	Line Chart
19	Female	20 - 29 years	Master’s degree	Yes	Yes	Column Chart	Column Chart	Column Chart	Column Chart

20	Female	20 - 29 years	Bachelor’s degree	Don’t exercise any professional activity	Yes	Line Chart	Line Chart	Line Chart	Line Chart
21	Female	20 - 29 years	High School	No	No	Area Chart	Table	Line Chart	Column Chart
22	Female	20 - 29 years	Master’s degree	No	Yes	Area Chart	Line Chart	Line Chart	Line Chart
23	Female	20 - 29 years	Bachelor’s degree	No	Yes	Area Chart	Line Chart	Line Chart	Line Chart
24	Male	40 - 49 years	Techno-professional School	No	Yes	Area Chart	Area Chart	Area Chart	Area Chart
25	Female	20 - 29 years	Master’s degree	No	Yes	Line Chart	Column Chart	Column Chart	Column Chart
26	Male	20 - 29 years	Master’s degree	No	No	Line Chart	Line Chart	Table	Table
27	Female	0 - 19 years	High School	Don’t exercise any professional activity	No	Area Chart	Line Chart	Line Chart	Line Chart
28	Female	20 - 29 years	Master’s degree	No	No	Area Chart	Table	Line Chart	Table
29	Female	20 - 29 years	High School	Don’t exercise any professional activity	Yes	Area Chart	Column Chart	Area Chart	Column Chart
30	Female	20 - 29 years	Bachelor’s degree	Yes	Yes	Line Chart	Line Chart	Column Chart	Column Chart
31	Female	50 - 59 years	Bachelor’s degree	No	No	Table	Line Chart	Line Chart	Column Chart
32	Female	20 - 29 years	Master’s degree	Yes	Yes	Line Chart	Column Chart	Area Chart	Column Chart
33	Female	20 - 29 years	Master’s degree	No	No	Line Chart	Line Chart	Line Chart	Line Chart
34	Female	20 - 29 years	Bachelor’s degree	Yes	Yes	Table	Column Chart	Table	Column Chart
35	Female	20 - 29 years	Bachelor’s degree	Yes	Yes	Area Chart	Column Chart	Line Chart	Column Chart
36	Female	20 - 29 years	Bachelor’s degree	Yes	Yes	Table	Line Chart	Line Chart	Line Chart
37	Female	20 - 29 years	High School	Don’t exercise any professional activity	No	Area Chart	Line Chart	Area Chart	Line Chart
38	Male	20 - 29 years	Bachelor’s degree	Don’t exercise any professional activity	Yes	Line Chart	Column Chart	Area Chart	Column Chart
39	Female	20 - 29 years	Master’s degree	No	Yes	Table	Column Chart	Table	Column Chart
40	Female	20 - 29 years	Bachelor’s degree	Yes	No	Line Chart	Column Chart	Table	Table
41	Female	20 - 29 years	Bachelor’s degree	Yes	No	Line Chart	Column Chart	Line Chart	Line Chart
42	Female	20 - 29 years	Bachelor’s degree	Yes	No	Table	Line Chart	Column Chart	Line Chart

43	Female	50 - 59 years	Bachelor's degree	No	No	Table	Table	Line Chart	Line Chart
44	Female	20 - 29 years	High School	Yes	No	Line Chart	Line Chart	Area Chart	Area Chart
45	Female	20 - 29 years	Master's degree	Yes	Yes	Area Chart	Column Chart	Column Chart	Column Chart
46	Female	20 - 29 years	Bachelor's degree	Yes	Yes	Column Chart	Column Chart	Column Chart	Column Chart
47	Female	0 - 19 years	High School	Don't exercise any profession al activity	No	Line Chart	Column Chart	Line Chart	Line Chart
48	Female	20 - 29 years	Master's degree	No	Yes	Line Chart	Line Chart	Column Chart	Column Chart
49	Male	20 - 29 years	Master's degree	Yes	Yes	Line Chart	Line Chart	Area Chart	Column Chart
50	Female	20 - 29 years	Bachelor's degree	No	No	Line Chart	Column Chart	Table	Column Chart
51	Male	20 - 29 years	Master's degree	Yes	Yes	Column Chart	Area Chart	Line Chart	Line Chart
52	Female	50 - 59 years	Techno-profession al School	Yes	No	Column Chart	Column Chart	Line Chart	Column Chart
53	Male	20 - 29 years	Master's degree	No	No	Column Chart	Line Chart	Line Chart	Line Chart
54	Female	20 - 29 years	Bachelor's degree	No	No	Area Chart	Column Chart	Line Chart	Line Chart
55	Female	20 - 29 years	Bachelor's degree	No	Yes	Table	Line Chart	Line Chart	Column Chart
56	Male	20 - 29 years	Master's degree	No	Yes	Line Chart	Line Chart	Column Chart	Column Chart
57	Female	20 - 29 years	Bachelor's degree	Yes	Yes	Column Chart	Column Chart	Column Chart	Line Chart
58	Male	20 - 29 years	Master's degree	No	No	Line Chart	Column Chart	Area Chart	Area Chart
59	Female	20 - 29 years	Master's degree	No	No	Column Chart	Table	Column Chart	Column Chart
60	Female	20 - 29 years	Master's degree	Don't exercise any profession al activity	Yes	Line Chart	Column Chart	Area Chart	Line Chart
61	Male	20 - 29 years	High School	No	No	Column Chart	Line Chart	Line Chart	Column Chart
62	Female	20 - 29 years	High School	Don't exercise any profession al activity	No	Table	Table	Column Chart	Table
63	Female	40 - 49 years	Master's degree	No	Yes	Line Chart	Table	Line Chart	Table
64	Female	0 - 19 years	Bachelor's degree	Yes	No	Line Chart	Line Chart	Line Chart	Line Chart
65	Male	20 - 29 years	Bachelor's degree	No	No	Line Chart	Line Chart	Line Chart	Area Chart
66	Male	20 - 29 years	Bachelor's degree	No	No	Line Chart	Area Chart	Line Chart	Column Chart
67	Male	20 - 29 years	Bachelor's degree	Don't exercise any profession al activity	Yes	Column Chart	Line Chart	Area Chart	Area Chart

68	Male	20 - 29 years	Techno-profession al School	No	No	Column Chart	Table	Line Chart	Table
69	Female	20 - 29 years	Bachelor's degree	Yes	Yes	Line Chart	Line Chart	Line Chart	Column Chart
70	Female	20 - 29 years	Master's degree	No	No	Area Chart	Area Chart	Line Chart	Column Chart
71	Male	20 - 29 years	Techno-profession al School	No	No	Table	Column Chart	Line Chart	Column Chart
72	Female	20 - 29 years	Master's degree	Don't exercise any profession al activity	Yes	Area Chart	Column Chart	Line Chart	Column Chart

**№**	**Questio n 5**	**Questio n 6**	**Question 7**	**Question 8**	**Question 9**	**Question 10**	**Questio n 11**	**Question 12**
1	Line Chart	Line Chart	Pie Chart	Donut Chart	Column Chart	Stacked Column Chart	Choropl eth map	A risk is constant within administrative limit
2	Area Chart	Line Chart	Bar Chart	Bar Chart	Column Chart	Two-sided Bar Chart	Choropl eth map	A risk is continuous in the space
3	Table	Table	Pie Chart	Pie Chart	Table	Table	Choropl eth map	A risk is continuous in the space
4	Stacked Column Chart	Column Chart	Column Chart	Column Chart	Table	Grouped Column Chart	Choropl eth map	A risk is constant within administrative limit
5	Line Chart	Line Chart	Column Chart	Donut Chart	Column Chart	Stacked Column Chart	Choropl eth map	A risk is constant within administrative limit
6	Area Chart	Stacked Column Chart	Pie Chart	Pie Chart	Column Chart	Two-sided Bar Chart	Choropl eth map	A risk is constant within administrative limit
7	Table	Column Chart	Pie Chart	100% Stacked Bar Chart	Donut Chart	Two-sided Bar Chart	Choropl eth map	A risk is constant within administrative limit
8	Stacked Column Chart	Stacked Column Chart	100% Stacked Bar Chart	Pie Chart	Pie Chart	Two-sided Bar Chart	Bubble map	A risk is constant within administrative limit
9	Stacked Column Chart	Line Chart	Donut Chart	Donut Chart	Pie Chart	Two-sided Bar Chart	Choropl eth map	A risk is continuous in the space
10	Table	Column Chart	Column Chart	Column Chart	Column Chart	Two-sided Bar Chart	Choropl eth map	A risk is continuous in the space
11	Line Chart	Stacked Column Chart	Donut Chart	100% Stacked Bar Chart	Donut Chart	Two-sided Bar Chart	Dasyme tric map	A risk is constant within administrative limit
12	Line Chart	Line Chart	Column Chart	Column Chart	Column Chart	Grouped Column Chart	Dasyme tric map	A risk is constant within administrative limit

13	Table	Table	Donut Chart	Pie Chart	Donut Chart	Stacked Column Chart a 100%	Chloropl eth map	A risk is constant within administrative limit
14	Area Chart	Column Chart	Pie Chart	Pie Chart	Donut Chart	Two-sided Bar Chart	Dasyme tric map	A risk is constant within administrative limit
15	Table	Column Chart	Bar Chart	Pie Chart	Column Chart	Two-sided Bar Chart	Choropl eth map	A risk is constant within administrative limit
16	Line Chart	Column Chart	Bar Chart	Pie Chart	Bar Chart	Two-sided Bar Chart	Dasyme tric map	A risk is continuous in the space
17	Area Chart	Line Chart	Column Chart	Pie Chart	Pie Chart	Two-sided Bar Chart	Bubble map	A risk is continuous in the space
18	Column Chart	Column Chart	Bar Chart	Column Chart	Bar Chart	Two-sided Bar Chart	Bubble map	A risk is constant within administrative limit
19	Column Chart	Column Chart	Column Chart	Pie Chart	Bar Chart	Two-sided Bar Chart	Choropl eth map	A risk is continuous in the space
20	Line Chart	Line Chart	Bar Chart	Donut Chart	Bar Chart	Two-sided Bar Chart	Dasyme tric map	A risk is continuous in the space
21	Line Chart	Table	Column Chart	Pie Chart	Table	Table	Bubble map	A risk is constant within administrative limit
22	Line Chart	Line Chart	Table	Pie Chart	Pie Chart	Two-sided Bar Chart	Choropl eth map	A risk is constant within administrative limit
23	Line Chart	Area Chart	Bar Chart	Pie Chart	Bar Chart	Two-sided Bar Chart	Choropl eth map	A risk is constant within administrative limit
24	Stacked Column Chart	Area Chart	Pie Chart	Pie Chart	Pie Chart	Two-sided Bar Chart	Bubble map	A risk is continuous in the space
25	Area Chart	Column Chart	Pie Chart	Pie Chart	Column Chart	Stacked Column Chart a 100%	Choropl eth map	A risk is constant within administrative limit
26	Line Chart	Line Chart	Pie Chart	Pie Chart	Pie Chart	Two-sided Bar Chart	Choropl eth map	A risk is constant within administrative limit
27	Line Chart	Line Chart	Bar Chart	Pie Chart	Bar Chart	Two-sided Bar Chart	Bubble map	A risk is constant within administrative limit
28	Column Chart	Table	Column Chart	Pie Chart	Column Chart	Two-sided Bar Chart	Choropl eth map	A risk is constant within administrative limit
29	Table	Column Chart	100% Stacked Bar Chart	Column Chart	Column Chart	Table	Choropl eth map	A risk is constant within administrative limit

30	Area Chart	Area Chart	Pie Chart	Pie Chart	Pie Chart	Two-sided Bar Chart	Choropl eth map	A risk is constant within administrative limit
31	Area Chart	Line Chart	Pie Chart	Pie Chart	Column Chart	Grouped Column Chart	Choropl eth map	A risk is constant within administrative limit
32	Line Chart	Stacked Column Chart	Bar Chart	Pie Chart	Pie Chart	Two-sided Bar Chart	Bubble map	A risk is constant within administrative limit
33	Stacked Column Chart	Stacked Column Chart	Pie Chart	Pie Chart	Table	Table	Choropl eth map	A risk is constant within administrative limit
34	Table	Column Chart	Pie Chart	Pie Chart	Pie Chart	Two-sided Bar Chart	Bubble map	A risk is continuous in the space
35	Stacked Column Chart	Column Chart	Bar Chart	Pie Chart	Bar Chart	Grouped Column Chart	Choropl eth map	A risk is constant within administrative limit
36	Area Chart	Stacked Column Chart	Pie Chart	Column Chart	Column Chart	Table	Choropl eth map	A risk is continuous in the space
37	Line Chart	Line Chart	Column Chart	Column Chart	Column Chart	Two-sided Bar Chart	Dasyme tric map	A risk is continuous in the space
38	Line Chart	Column Chart	Pie Chart	Column Chart	Pie Chart	Grouped Column Chart	Bubble map	A risk is continuous in the space
39	Table	Column Chart	Pie Chart	Pie Chart	Bar Chart	Two-sided Bar Chart	Choropl eth map	A risk is constant within administrative limit
40	Table	Stacked Column Chart	Bar Chart	Bar Chart	Bar Chart	Two-sided Bar Chart	Choropl eth map	A risk is constant within administrative limit
41	Table	Column Chart	Pie Chart	Donut Chart	Bar Chart	Grouped Column Chart	Choropl eth map	A risk is constant within administrative limit
42	Table	Table	Column Chart	Pie Chart	Column Chart	Grouped Column Chart	Choropl eth map	A risk is constant within administrative limit
43	Table	Table	Table	Table	Table	Table	Choropl eth map	A risk is constant within administrative limit
44	Line Chart	Line Chart	Column Chart	Pie Chart	Column Chart	Two-sided Bar Chart	Choropl eth map	A risk is continuous in the space
45	Table	Line Chart	Pie Chart	100% Stacked Bar Chart	Pie Chart	Two-sided Bar Chart	Choropl eth map	A risk is constant within administrative limit
46	Stacked Column Chart	Column Chart	Bar Chart	Table	Bar Chart	Grouped Column Chart	Bubble map	A risk is continuous in the space

47	Table	Stacked Column Chart	Pie Chart	Column Chart	Column Chart	Two-sided Bar Chart	Bubble map	A risk is constant within administrative limit
48	Stacked Column Chart	Column Chart	Bar Chart	Pie Chart	Table	Two-sided Bar Chart	Choropl eth map	A risk is constant within administrative limit
49	Line Chart	Stacked Column Chart	Bar Chart	Table	Column Chart	Two-sided Bar Chart	Choropl eth map	A risk is continuous in the space
50	Table	Table	Bar Chart	Bar Chart	Column Chart	Two-sided Bar Chart	Dasyme tric map	A risk is constant within administrative limit
51	Line Chart	Line Chart	Bar Chart	Pie Chart	Pie Chart	Two-sided Bar Chart	Choropl eth map	A risk is constant within administrative limit
52	Area Chart	Column Chart	Pie Chart	Table	Table	Stacked Column Chart a 100%	Bubble map	A risk is constant within administrative limit
53	Stacked Column Chart	Stacked Column Chart	Column Chart	Pie Chart	Column Chart	Two-sided Bar Chart	Choropl eth map	A risk is constant within administrative limit
54	Area Chart	Area Chart	Pie Chart	Pie Chart	Table	Grouped Column Chart	Bubble map	A risk is constant within administrative limit
55	Table	Table	Pie Chart	Pie Chart	Bar Chart	Two-sided Bar Chart	Bubble map	A risk is constant within administrative limit
56	Stacked Column Chart	Column Chart	Column Chart	Pie Chart	100% Stacked Bar Chart	Two-sided Bar Chart	Choropl eth map	A risk is constant within administrative limit
57	Stacked Column Chart	Column Chart	Pie Chart	Donut Chart	Pie Chart	Two-sided Bar Chart	Choropl eth map	A risk is constant within administrative limit
58	Area Chart	Column Chart	Column Chart	Donut Chart	Bar Chart	Two-sided Bar Chart	Bubble map	A risk is continuous in the space
59	Line Chart	Table	Table	Pie Chart	Column Chart	Two-sided Bar Chart	Bubble map	A risk is constant within administrative limit
60	Line Chart	Line Chart	Column Chart	Donut Chart	Pie Chart	Two-sided Bar Chart	Choropl eth map	A risk is constant within administrative limit
61	Line Chart	Column Chart	Column Chart	Pie Chart	Donut Chart	Two-sided Bar Chart	Choropl eth map	A risk is constant within administrative limit
62	Table	Column Chart	Pie Chart	Pie Chart	Table	Grouped Column Chart	Choropl eth map	A risk is constant within administrative limit

63	Stacked Column Chart	Stacked Column Chart	Column Chart	Pie Chart	Table	Two-sided Bar Chart	Choropleth map	A risk is constant within administrative limit
64	Table	Column Chart	Pie Chart	Table	Column Chart	Two-sided Bar Chart	Choropleth map	A risk is constant within administrative limit
65	Line Chart	Line Chart	Column Chart	Donut Chart	Column Chart	Grouped Column Chart	Bubble map	A risk is continuous in the space
66	Area Chart	Stacked Column Chart	Pie Chart	Pie Chart	Bar Chart	Two-sided Bar Chart	Choropleth map	A risk is constant within administrative limit
67	Line Chart	Line Chart	Pie Chart	Donut Chart	Column Chart	Stacked Column Chart	Choropleth map	A risk is constant within administrative limit
68	Table	Table	Pie Chart	Pie Chart	Table	Table	Bubble map	A risk is continuous in the space
69	Stacked Column Chart	Column Chart	Column Chart	Pie Chart	Column Chart	Stacked Column Chart	Choropleth map	A risk is continuous in the space
70	Area Chart	Area Chart	Pie Chart	Pie Chart	Donut Chart	Grouped Column Chart	Choropleth map	A risk is constant within administrative limit
71	Table	Stacked Column Chart	Bar Chart	Pie Chart	Bar Chart	Grouped Column Chart	Bubble map	A risk is continuous in the space
72	Stacked Column Chart	Area Chart	Bar Chart	Pie Chart	Pie Chart	Two-sided Bar Chart	Choropleth map	A risk is constant within administrative limit

Table S2. Questionnaire responses for the second round.

**Nº**	**Gender**	**Age group**	**Highest education level**	**Have the professional activity related with health sector**	**Are familiar with COVID-19 dashboards**	**Question 1**	**Question 2**	**Question 3**	**Question 4**
1	Male	20 a 29 anos	Bachelor's degree	Don't exercise any professional activity	Yes	Column Chart	Line Chart	Line Chart	Line Chart
2	Female	20 a 29 anos	Master's degree	Yes	Yes	Area Chart	Table	Line Chart	Table
3	Female	20 a 29 anos	Master's degree	Yes	No	Area Chart	Line Chart	Line Chart	Line Chart

4	Female	20 a 29 anos	Master's degree	No	No	Line Chart	Column Chart	Line Chart	Area Chart
5	Male	20 a 29 anos	Bachelor's degree	No	Yes	Column Chart	Table	Line Chart	Column Chart
6	Male	20 a 29 anos	High School	No	No	Area Chart	Line Chart	Column Chart	Column Chart
7	Female	20 a 29 anos	Techno-professional School	No	Yes	Column Chart	Column Chart	Column Chart	Column Chart
8	Male	20 a 29 anos	Master's degree	Yes	Yes	Line Chart	Table	Line Chart	Column Chart
9	Male	20 a 29 anos	Bachelor's degree	No	Yes	Line Chart	Line Chart	Line Chart	Line Chart
10	Male	30 a 39 anos	Master's degree	No	Yes	Line Chart	Table	Line Chart	Table
11	Female	0 a 19 anos	High School	Don't exercise any professional activity	No	Line Chart	Line Chart	Line Chart	Column Chart
12	Female	20 a 29 anos	Bachelor's degree	Yes	Yes	Line Chart	Column Chart	Line Chart	Column Chart
13	Female	20 a 29 anos	Master's degree	No	Yes	Area Chart	Column Chart	Area Chart	Area Chart
14	Male	20 a 29 anos	Master's degree	No	Yes	Area Chart	Column Chart	Line Chart	Line Chart
15	Male	20 a 29 anos	Bachelor's degree	Don't exercise any professional activity	Yes	Line Chart	Line Chart	Line Chart	Column Chart
16	Female	20 a 29 anos	Master's degree	Yes	Yes	Column Chart	Column Chart	Column Chart	Column Chart
17	Female	20 a 29 anos	Bachelor's degree	Don't exercise any professional activity	Yes	Line Chart	Column Chart	Line Chart	Column Chart
18	Female	20 a 29 anos	High School	No	No	Line Chart	Table	Line Chart	Column Chart
19	Female	20 a 29 anos	Master's degree	No	Yes	Line Chart	Line Chart	Line Chart	Column Chart
20	Female	20 a 29 anos	Master's degree	No	Yes	Line Chart	Column Chart	Column Chart	Column Chart
21	Male	20 a 29 anos	Master's degree	No	No	Line Chart	Line Chart	Table	Table
22	Female	0 a 19 anos	High School	Don't exercise any professional activity	No	Area Chart	Line Chart	Line Chart	Line Chart
23	Female	20 a 29 anos	Master's degree	No	No	Table	Table	Line Chart	Table
24	Female	20 a 29 anos	High School	Don't exercise any professio	Yes	Area Chart	Column Chart	Area Chart	Column Chart

				nal activity					
25	Female	50 a 59 anos	Bachelor's degree	No	No	Line Chart	Line Chart	Line Chart	Column Chart
26	Female	20 a 29 anos	Bachelor's degree	Yes	Yes	Area Chart	Column Chart	Line Chart	Column Chart
27	Female	20 a 29 anos	Bachelor's degree	Yes	Yes	Line Chart	Line Chart	Line Chart	Column Chart
28	Female	20 a 29 anos	High School	Don’t exercise any professional activity	No	Area Chart	Line Chart	Area Chart	Column Chart
29	Male	20 a 29 anos	Bachelor's degree	Don’t exercise any professional activity	Yes	Line Chart	Column Chart	Area Chart	Column Chart
30	Female	20 a 29 anos	Master's degree	No	Yes	Table	Column Chart	Table	Column Chart
31	Female	20 a 29 anos	Bachelor's degree	Yes	No	Table	Line Chart	Line Chart	Column Chart
32	Female	20 a 29 anos	High School	Yes	No	Line Chart	Column Chart	Line Chart	Column Chart
33	Female	20 a 29 anos	Master's degree	Yes	Yes	Column Chart	Column Chart	Area Chart	Column Chart
34	Female	20 a 29 anos	Bachelor's degree	Yes	Yes	Column Chart	Column Chart	Column Chart	Column Chart
35	Female	20 a 29 anos	Master's degree	No	Yes	Column Chart	Column Chart	Line Chart	Column Chart
36	Male	20 a 29 anos	Master's degree	Yes	Yes	Line Chart	Column Chart	Area Chart	Column Chart
37	Male	20 a 29 anos	Master's degree	Yes	Yes	Column Chart	Area Chart	Line Chart	Line Chart
38	Male	20 a 29 anos	Master's degree	No	No	Column Chart	Line Chart	Line Chart	Line Chart
39	Male	20 a 29 anos	Master's degree	No	Yes	Line Chart	Line Chart	Column Chart	Column Chart
40	Female	20 a 29 anos	Bachelor's degree	Yes	Yes	Column Chart	Line Chart	Line Chart	Line Chart
41	Male	20 a 29 anos	Master's degree	No	No	Line Chart	Column Chart	Area Chart	Area Chart
42	Female	20 a 29 anos	Master's degree	Don’t exercise any professional activity	Yes	Line Chart	Column Chart	Area Chart	Line Chart
43	Female	20 a 29 anos	High School	Don’t exercise any professional activity	No	Table	Table	Table	Table
44	Male	20 a 29 anos	Bachelor's degree	No	No	Line Chart	Column Chart	Line Chart	Line Chart
45	Male	20 a 29 anos	Bachelor's degree	No	No	Line Chart	Column Chart	Line Chart	Column Chart
46	Male	20 a 29 anos	Bachelor's degree	Don’t exercise any	Yes	Column Chart	Column Chart	Line Chart	Column Chart

				professional activity					
47	Male	20 a 29 anos	Techno-professional School	No	No	Column Chart	Table	Line Chart	Column Chart

Nº	Question 5	Question 6	Question 7	Question 8	Question 9	Question 10	Question 11	Question 12
	Line Chart	Line Chart	Column Chart	Pie Chart	Column Chart	Two-sided Bar Chart	Choropleth map	A risk is constant within administrative limit
	Table	Table	Pie Chart	Pie Chart	Table	Table	Choropleth map	A risk is continuous in the space
	Line Chart	Line Chart	Pie Chart	Pie Chart	Column Chart	Stacked Column Chart	Choropleth map	A risk is constant within administrative limit
	Stacked Column Chart	Stacked Column Chart	Pie Chart	Pie Chart	Pie Chart	Two-sided Bar Chart	Choropleth map	A risk is constant within administrative limit
	Column Chart	Column Chart	Donut Chart	100% Stacked Bar Chart	Donut Chart	Two-sided Bar Chart	Choropleth map	A risk is constant within administrative limit
	Table	Column Chart	Donut Chart	Donut Chart	Pie Chart	Two-sided Bar Chart	Choropleth map	A risk is continuous in the space
	Column Chart	Column Chart	Column Chart	Column Chart	Column Chart	Two-sided Bar Chart	Choropleth map	A risk is continuous in the space
	Line Chart	Table	Pie Chart	Donut Chart	Donut Chart	Two-sided Bar Chart	Choropleth map	A risk is constant within administrative limit
	Line Chart	Line Chart	Column Chart	Column Chart	Column Chart	Two-sided Bar Chart	Choropleth map	A risk is constant within administrative limit
	Line Chart	Table	Bar Chart	Donut Chart	Donut Chart	Stacked Column Chart	Choropleth map	A risk is constant within administrative limit
	Line Chart	Column Chart	Pie Chart	Pie Chart	Bar Chart	Two-sided Bar Chart	Choropleth map	A risk is constant within administrative limit
	Table	Column Chart	Bar Chart	Pie Chart	Column Chart	Two-sided Bar Chart	Choropleth map	A risk is constant

							within administrative limit
Line Chart	Column Chart	Bar Chart	Pie Chart	Bar Chart	Two-sided Bar Chart	Dasymetric map	A risk is continuous in the space
Area Chart	Line Chart	Column Chart	Pie Chart	Column Chart	Two-sided Bar Chart	Bubble map	A risk is constant within administrative limit
Stacked Column Chart	Column Chart	Column Chart	Pie Chart	Column Chart	Two-sided Bar Chart	Choropleth map	A risk is constant within administrative limit
Column Chart	Column Chart	Column Chart	Pie Chart	Column Chart	Two-sided Bar Chart	Choropleth map	A risk is continuous in the space
Line Chart	Column Chart	Bar Chart	Pie Chart	Column Chart	Two-sided Bar Chart	Choropleth map	A risk is constant within administrative limit
Line Chart	Table	Column Chart	Pie Chart	Column Chart	Two-sided Bar Chart	Bubble map	A risk is constant within administrative limit
Line Chart	Line Chart	Bar Chart	Pie Chart	Bar Chart	Two-sided Bar Chart	Choropleth map	A risk is constant within administrative limit
Area Chart	Column Chart	Pie Chart	Pie Chart	Pie Chart	Stacked Column Chart	Choropleth map	A risk is constant within administrative limit
Line Chart	Line Chart	Pie Chart	Pie Chart	Pie Chart	Two-sided Bar Chart	Choropleth map	A risk is constant within administrative limit
Line Chart	Line Chart	Bar Chart	Pie Chart	Bar Chart	Two-sided Bar Chart	Choropleth map	A risk is constant within administrative limit
Column Chart	Table	Column Chart	Pie Chart	Column Chart	Two-sided Bar Chart	Choropleth map	A risk is constant within administrative limit
Table	Column Chart	Bar Chart	Pie Chart	Column Chart	Table	Choropleth map	A risk is constant within administrative limit
Table	Line Chart	Pie Chart	Pie Chart	Column Chart	Two-sided Bar Chart	Choropleth map	A risk is constant

							within administrative limit
Stacked Column Chart	Column Chart	Bar Chart	Pie Chart	Bar Chart	Two-sided Bar Chart	Choropleth map	A risk is constant within administrative limit
Line Chart	Column Chart	Column Chart	Pie Chart	Column Chart	Two-sided Bar Chart	Choropleth map	A risk is constant within administrative limit
Line Chart	Line Chart	Column Chart	Column Chart	Column Chart	Two-sided Bar Chart	Dasymetric map	A risk is continuous in the space
Line Chart	Column Chart	Pie Chart	Pie Chart	Pie Chart	Two-sided Bar Chart	Bubble map	A risk is constant within administrative limit
Table	Column Chart	Pie Chart	Pie Chart	Bar Chart	Two-sided Bar Chart	Choropleth map	A risk is constant within administrative limit
Column Chart	Column Chart	Column Chart	Pie Chart	Column Chart	Grouped Column Chart	Choropleth map	A risk is constant within administrative limit
Line Chart	Column Chart	Column Chart	Pie Chart	Column Chart	Two-sided Bar Chart	Choropleth map	A risk is constant within administrative limit
Table	Area Chart	Bar Chart	100% Stacked Bar Chart	Pie Chart	Two-sided Bar Chart	Choropleth map	A risk is constant within administrative limit
Stacked Column Chart	Column Chart	Bar Chart	Table	Bar Chart	Grouped Column Chart	Bubble map	A risk is constant within administrative limit
Stacked Column Chart	Column Chart	Bar Chart	Pie Chart	Table	Two-sided Bar Chart	Choropleth map	A risk is constant within administrative limit
Line Chart	Stacked Column Chart	Column Chart	Pie Chart	Column Chart	Two-sided Bar Chart	Choropleth map	A risk is constant within administrative limit
Line Chart	Line Chart	Bar Chart	Pie Chart	Pie Chart	Two-sided Bar Chart	Choropleth map	A risk is constant within administrative limit

Stacked Column Chart	Stacked Column Chart	Column Chart	Pie Chart	Column Chart	Two-sided Bar Chart	Choropleth map	A risk is constant within administrative limit
Stacked Column Chart	Column Chart	Column Chart	Pie Chart	Column Chart	Two-sided Bar Chart	Choropleth map	A risk is constant within administrative limit
Stacked Column Chart	Column Chart	Pie Chart	Donut Chart	Pie Chart	Two-sided Bar Chart	Choropleth map	A risk is constant within administrative limit
Area Chart	Column Chart	Column Chart	Donut Chart	Bar Chart	Two-sided Bar Chart	Bubble map	A risk is continuous in the space
Line Chart	Line Chart	Column Chart	Pie Chart	Column Chart	Two-sided Bar Chart	Choropleth map	A risk is constant within administrative limit
Table	Column Chart	Pie Chart	Pie Chart	Pie Chart	Grouped Column Chart	Choropleth map	A risk is constant within administrative limit
Line Chart	Column Chart	Column Chart	Donut Chart	Column Chart	Grouped Column Chart	Bubble map	A risk is continuous in the space
Line Chart	Column Chart	Pie Chart	Pie Chart	Column Chart	Two-sided Bar Chart	Choropleth map	A risk is constant within administrative limit
Line Chart	Line Chart	Column Chart	Pie Chart	Column Chart	Two-sided Bar Chart	Choropleth map	A risk is constant within administrative limit
Table	Table	Pie Chart	Pie Chart	Table	Table	Bubble map	A risk is continuous in the space
